# Occupational Lead Exposure and Brain Tumors: Systematic Review and Meta-Analysis

**DOI:** 10.3390/ijerph17113975

**Published:** 2020-06-03

**Authors:** Joonho Ahn, Mi Yeon Park, Mo-Yeol Kang, In-Soo Shin, Sungjae An, Hyoung-Ryoul Kim

**Affiliations:** 1 Department of Occupational and Environmental Medicine, Seoul St. Mary’s Hospital, College of Medicine, The Catholic University of Korea, Seoul 06591, Korea; drcox@naver.com (J.A.); snaptoon@naver.com (M.-Y.K.); 2 Department of Radiology, University of Ulsan, College of Medicine, Asan Medical Center, Seoul 05505, Korea; lpk1102@gmail.com; 3 Graduate School of Education, Dongguk University, Seoul 04620, Korea; 9065031@hanmail.net; 4 Department of Neurosurgery, Seoul National University, Bundang Hospital, Seongnam 13620, Korea; annuguri@hotmail.com

**Keywords:** brain tumor, lead compound, carcinogen, meta-analysis

## Abstract

(1) *Background*: Due to inconsistencies in epidemiological findings, there has been uncertainty regarding the association of lead compounds with brain tumors. We performed a meta-analysis of published case-control and cohort studies exploring lead compound exposure and brain tumor risk. (2) *Methods*: We searched PubMed, Embase^®^, and Cochrane to find eligible studies. Eighteen studies were selected for assessment of occupational exposure to lead compound and brain tumor. Pooled estimates of odds ratios (ORs) were obtained using random effects models. We assessed the differences through subgroup analysis according to tumor type, study design, measurements of exposure, and tumor outcome. Statistical tests for publication bias, heterogeneity, and sensitivity analysis were applied. (3) *Results*: Our systematic review and meta-analysis showed a not significant association with lead exposure and risk of benign and malignant brain tumors (pooled OR = 1.11, 95% Confidence Interval (CI): 0.95–1.29). Including only malignant brain tumors, the risk of brain tumor was significantly increased (pooled OR = 1.13, 95% CI: 1.04–1.24). (4) *Conclusions*: This meta-analysis provides suggestive evidence for an association between lead compound exposure and brain tumor. In future studies, it will be necessary to identify the effect of lead compounds according to the types of brain tumor.

## 1. Introduction

Primary brain and central nervous system (CNS) tumors are uncommon but are among the most feared diseases. These tumors cause severe, complex symptoms and are usually incurable, seriously affecting the patient’s quality of life. The global age-standardized rate of brain and CNS cancers is 3.5 per 100,000 (3.9 for males and 3.1 for females); the global age-standardized mortality for primary malignant brain and CNS tumors is 3.2 for males and 2.3 for females per 100,000 [1]. Brain tumors can be divided into benign and malignant tumors on the basis of their behavior. They can also be classified on the basis of histology into such groups as diffuse astrocytic and oligodendroglial tumors, other gliomas, ependymal tumors, meningiomas, germ cell tumors, and tumors of the cranial and paraspinal nerves (including schwannoma) [2]. The most common types of primary brain tumors are glioblastomas (of diffuse astrocytic and oligodendroglial tumors) and meningiomas [3]. The etiology of brain tumors is not well understood. Several factors known to cause brain tumors include ionizing radiation exposure, electromagnetic fields, and certain hereditary syndromes such as neurofibromatosis [3,4,5,6].

Lead has been defined as a “probable human carcinogen” by the International Agency for Research on Cancer (IARC) and as a compound that is “reasonably anticipated” to be human carcinogen by the National Toxicology Programme (NTP), primarily based on lung and stomach cancers, but lead is also known to have some suggestive carcinogenic effects on kidney and brain [5,6]. A recent review article titled “Research Recommendations for Selected IARC-Classified Agents” recommended further studies about the relationship of several cancers including brain cancer with lead exposure [7].

Lead is a well-known neurotoxin, especially in early childhood. Lead exposure to the developing nervous system can be more toxic than on a mature brain and can affect mental development and intelligence and have long-term consequences [8]. In the aging brain, lead can also cause neurodegenerative diseases like Alzheimer’s disease or Parkinson disease [9]. The mechanisms of lead-induced neurotoxicity are complex, caused by oxidative stress, membrane physics alteration, and impairment of neurotransmission [8]. In contrast, the relationship of lead exposure and brain tumor is not well known.

There have been some cohort and case-control studies about the relationship between lead exposure and brain tumors. However, the results of these studies have been inconsistent. A study by Steenland et al. (2019) showed significant positive trends for malignant brain tumors with increasing lead exposure [10]. On the other hand, Bhatti et al. reported that there was no association between lead exposure and glioma [11]. Twenty years ago, in a previous meta-analysis by Steenland and Boffetta, a high level of lead exposure was not significantly associated with increased risk of brain cancer (combined relative risk (R) = 1.06, CI: 0.81–1.40), unlike lung and stomach cancer [12]. However, the heterogeneity of brain and CNS tumors and the limitation of exposure assessment make it difficult to identify the exact association. Moreover, in that meta-analysis, no consideration was given to malignancy of brain tumor or research design. In another recent meta-analysis by Meng et al., the authors argue that lead is a risk factor for meningiomas and brain cancer and a protective factor for gliomas [13], but they did not show a significant difference. That study had several limitations in research method. First, although a systematic review collects all studies related to a given topic and analyzes their results, Meng et al. did not collect all published studies [14,15]. Second, in meta-studies, researchers should take great care when using non-independent data. Although each study was mentioned as a result of follow-up, these non-independent studies were used carelessly (Bhatti et al. [11] and Rajaraman et al. [16]; Steenland et al. [17] and McElvenny et al. [18]). Finally, they did not consider whether the study outcome was incidence or mortality or whether the method of exposure measurement was blood lead level or job information.

To overcome the limitations of previous studies [13], we planned our systematic review and meta-analysis to include all available case-control and cohort studies in order to identify the association between lead exposure and brain or CNS tumors. In particular, we analyzed “all tumors” and “only malignant tumors” separately since it is clinically important to distinguish malignant tumors from benign. We also evaluated the relationship between brain tumors and lead exposure by adjusting the research design, the method of exposure assessment, the reference category, the sex, and the tumor type in subgroup analyses.

## 2. Materials and Methods

### 2.1. Data Sources and Search Strategy

We followed the reporting standards for systematic reviews and meta-analyses of observational studies according to the MOOSE (Meta-analysis of Observational Studies in Epidemiology) protocol [19]. Two trained librarians and two authors independently used the PubMed [20], Embase^®^ [21], and Cochrane [22] databases to conduct a comprehensive and systematic search for articles published by January 2020. We also performed a manual search, using the main keywords “Occupations”, “Work”, “Workplace”, “Lead”, “Metals, “Heavy”, “Brain Neoplasms”, “Glioma”, “Meningioma”, “Carcinoma”, and “Neoplasms” (Supplementary Material: Methods: Search strategies for PubMed, EMBASE, Cochrane database). Only articles published in English were considered. The full texts were obtained through manual retrieval, a document delivery service, and direct contacts with the authors.

### 2.2. Selection Criteria

Studies meeting the following eligibility criteria were included: (1) the study was of a case-control study or cohort study design; (2) the study population was adult workers; (3) the study reported the effect size of brain tumors in lead-exposed workers; (4) excluded were reviews, essays, conference abstracts, letters, and commentaries; (5) when multiple publications from the same study population were identified, we included the most thorough and recent article describing the most up-to-date data. In the case of errata, the results of the study were replaced with modified values.

### 2.3. Data Extraction and Rating Quality of Evidence

Two authors (J.A. and M.Y.P.) independently extracted relevant data from the studies, including authors, year of publication, sex, study period, study population, definitions and measurements of occupational lead exposure, effect size, and confidence interval of the outcome results. All cases of inconsistencies in the extracted data were discussed and resolved with the rest of the author team. When there was missing or unclear information in the published article, we contacted the author via email and requested the information.

Two authors (J.A. and M.Y.P.) independently conducted the risk of bias assessment of the included studies using the Newcastle–Ottawa scale [23]. Similar to a previous study [24], we categorized the studies by risk grade of bias, as low (7–9 stars), medium (5–6 stars), or high (0–4 stars). If there was any discrepancy in quality assessment, the rest of the author team discussed and resolved it.

### 2.4. Data Synthesis and Statistical Analysis

The primary meta-analysis was conducted to assess the association between occupational lead exposure and brain tumor. To calculate the pooled odds ratios (ORs), if the studies were heterogeneous both clinically and statistically, we used the random effect model [25]. Regarding brain tumor, what is important is whether they are malignant or benign. Therefore, we first assessed the association between occupational lead exposure and brain tumor, including both malignant and benign. The second analysis was limited to only malignant brain cancers.

We conducted subgroup analyses to examine the impact of using research design (case-control study vs cohort), the exposure assessment method (job information vs blood lead (Pb) level), outcome (brain tumor mortality vs incidence), and reference category (internal reference vs external reference). Additionally, in cases where the results were given according to tumor type and sex, we also performed subgroup analysis.

We calculated the effect size and conducted analyses based on Borenstein et al. [26]. Pooled estimates of ORs were obtained using random effects models. The three estimates—standardized mortality ratio, standardized incidence ratio, and hazard ratio—were treated as proxies for ORs, as in previous studies [27,28,29,30,31].

The risk of publication bias was determined by the funnel plot method and Begg’s test, which are appropriate when there are at least 10 studies included in a meta-analysis [32]. Using the “trim and fill” method, we explored the possible nature of the missing studies to estimate the true effect size for publication bias and sensitive analysis. Considering outliers, sensitivity analysis was also carried out after excluding those studies deemed to have high or medium risk of bias.

All meta-analyses were performed in R software (version 3.6.2; www.r-project.org), where the “meta” and “metaphor” packages were used to estimate the models and apply “trim and fill” and Begg’s test [33].

## 3. Results

### 3.1. Study Characteristics

**Figure 1:**
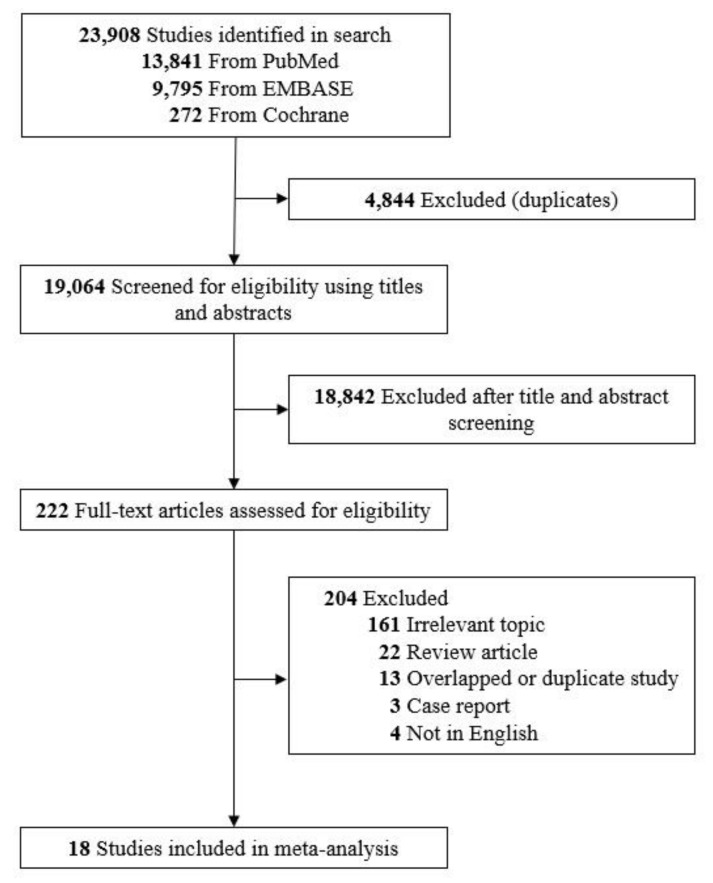
Flow diagram of study selection in the current systematic review and meta-analysis.

A total of 23,908 papers from the three chosen databases were identified by the initial search strategy. After removal of duplicates and title/abstract screening, 222 papers were selected for full-text screening. After we reviewed the full texts of the potentially eligible articles, 18 papers meeting the inclusion criteria were selected for the final analysis (Figure 1) [10,11,14,15,34,35,36,37,38,39,40,41,42,43,44,45,46,47].

The characteristics of the studies included are summarized in Table 1. Of the included studies, 7 [11,14,15,38,41,42,45] used case-control design and 11 [10,34,35,36,37,39,40,43,44,46,47] used cohort design. Occupational lead exposure was evaluated in two ways: measurements of blood lead level [10,14,43,47] and job information [11,15,34,35,36,37,38,39,40,41,42,44,45,46]. The reference categories also varied among the different studies: some adopted the non-exposed population (external reference) [35,36,37,39,43,44,46], and others adopted the lowest exposure categories (internal reference) [10,11,14,15,34,38,40,41,42,45,47]. Among the included studies, 13 evaluated the association between occupational lead exposure and brain tumor incidence [10,11,14,15,35,37,38,39,40,41,43,44,45]. Five assessed the association between occupational lead exposure and brain tumor mortality [34,36,42,46,47]. A total of 13 studies reported sex, 12 results [15,36,37,38,39,40,43,44,45,46,47] for men and 8 [15,35,38,40,42,45,46] for women. A total of nine studies were presented with tumor type, of which five results [10,11,14,41] were glioma, six [11,15,40,42,45] were meningioma, and one was acoustic neuroma [38]. Ten results were rated as having a low risk of bias [10,15,31,32,33,38,39,42,43], three results were rated as having a medium risk of bias [14,39,43], and 11 results were as having a high risk of bias [11,35,36,44,45,46].

### 3.2. Meta-Analysis on Brain Tumor (Both Malignant and Benign) and Occupational Lead Exposure

**Figure 2:**
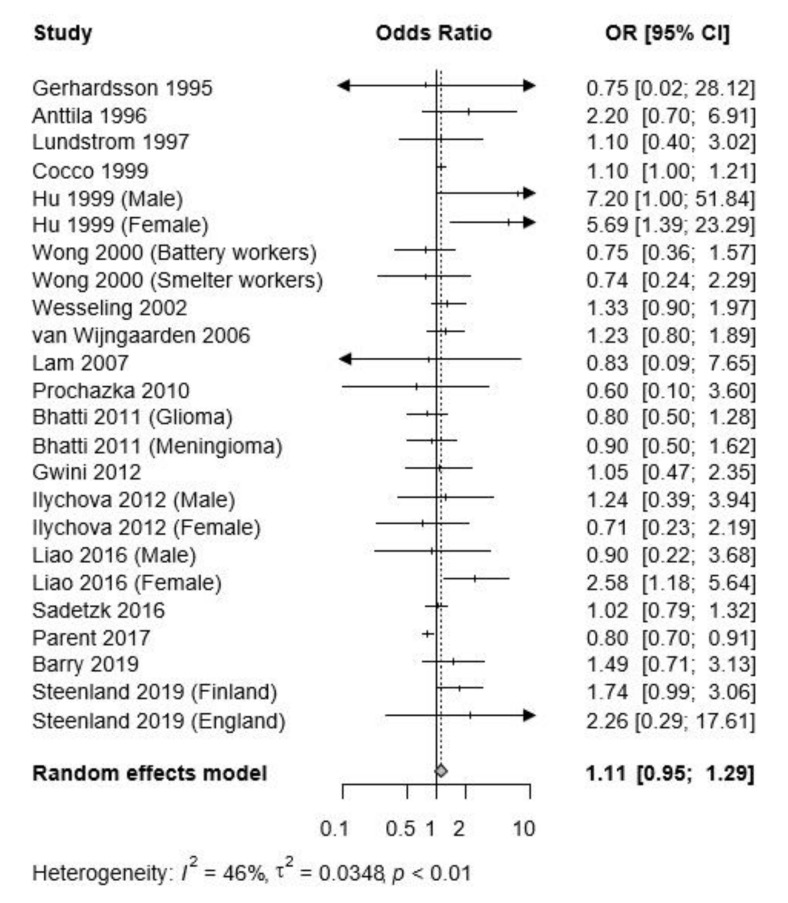
Forest plot of the risk estimates, log odds ratios (OR), and 95% confidence intervals (CI) from the studies included in the meta-analysis of the association between occupational lead exposure and all brain tumors.

The pooled OR for brain tumor (both malignant and benign) of occupational lead exposure was 1.11 (95% CI: 0.95–1.29) (Figure 2). Sensitivity analysis by excluding studies of high risk or studies of high and medium risk of bias did not modify the results of the meta-analysis (Figures S1 and S2).

Subgroup analyses were carried out (Table 2), and the differences in the subgroups, such as research design, tumor outcome, and reference category, were not statistically significant. However, the subgroup results by exposure assessment method appeared to be discordant. Studies using blood lead (Pb) level showed a significantly increased risk (pooled OR = 1.67, 95% CI: 1.12–2.49) (Table 2). However, studies using job information showed no significant improvement (pooled OR = 1.04, 95% CI: 0.89–1.23).

Begg’s test indicated no evidence of publication bias in the present study (*P*_Begg_ = 0.8817), nor was there evidence of publication bias observed on visual inspection of funnel plot asymmetry across all of the studies (Figure S3). The trim and fill method imputed three missing studies (Figure S3); after imputing them for publication bias, the pooled OR for brain tumor remained unchanged (pooled OR = 1.0468, 95% CI: 0.8860–1.2369).

### 3.3. Meta-Analysis on Brain Cancer (Only Malignant) and Occupational Lead Exposure

**Figure 3:**
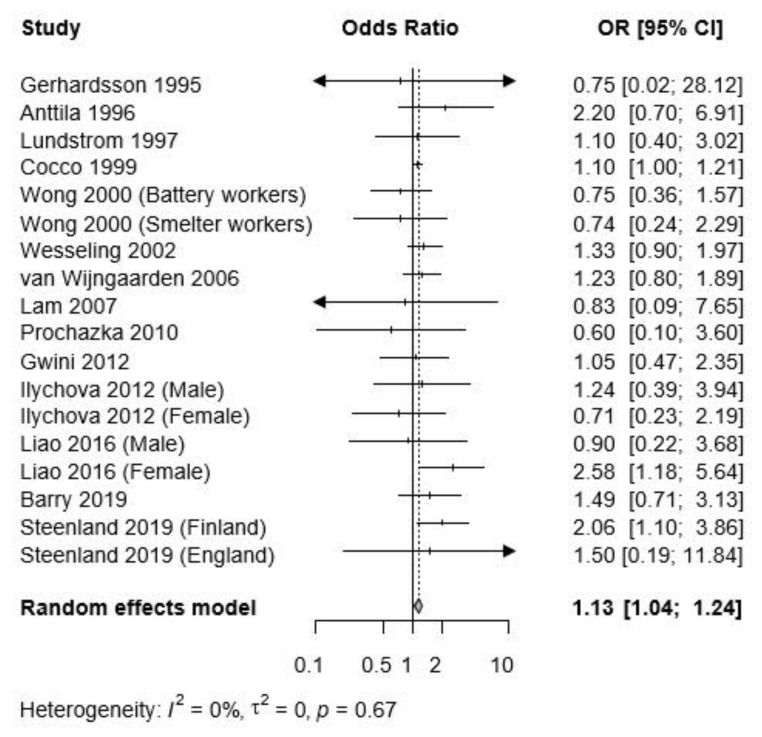
Forest plot of the risk estimates, log odds ratios (OR), and 95% confidence intervals (CI) from the studies included in the meta-analysis of the association between occupational lead exposure and malignant brain tumors.

When analysis was limited to only malignant brain cancers, there was a significantly increased risk for brain cancer associated with lead exposure (pooled OR = 1.13, 95% CI: 1.041.24), with homogeneity (*I*^2^ = 0%, *p* = 0.67) (Figure 3). Sensitivity analysis by excluding studies of high risk or studies of high and medium risk of bias also did not modify the results of the meta-analysis and showed robust results (Figures S4 and S5).

Similar to the meta-analysis on brain tumor, the subgroup results by research design and reference category did not show significant differences, while the subgroup results by exposure assessment method showed significant differences (Table 3).

Unlike the meta-analysis on brain tumor, the subgroup by cancer outcome also showed statistically significant differences. For brain cancer incidence, the effect size was larger than for brain cancer mortality (brain cancer incidence: pooled OR = 1.42, 95% CI: 1.09–1.85; brain cancer mortality: pooled OR = 1.10, 95% CI: 1.01–1.21).

Begg’s test indicated no evidence of publication bias in the present study (*P*_Begg_ = 0.5699). No evidence for publication bias was observed on visual inspection of funnel plot asymmetry across all of the studies (Figure S6). Furthermore, the trim and fill method did not impute any missing studies due to publication bias.

### 3.4. Subgroup Analysis on Tumor Type and Sex

Nine of the studies used specific tumor type, and 13 studies used specific sex. The subgroup result by sex did not show significant differences (Table S1). Although the subgroup result by sex also did not show significant differences, for meningioma, the effect size was larger than for glioma (meningioma: pooled OR = 1.69, 95% CI: 1.02–2.79; glioma: pooled OR = 1.03, 95% CI: 0.67–1.57).

## 4. Discussion

Occupational exposure to lead has been confirmed to be probably carcinogenic (group 2A) by the IARC. However, the evidence that lead exposure is associated with brain tumor has been inconsistent. Our systematic review and meta-analysis showed a significant association with lead exposure and risk of malignant brain tumors. Including all benign and malignant brain tumors, the risk of brain tumor was slightly increased to a statistically non-significant level.

### 4.1. Previous Study

Previous studies have suggested inconsistent results. Gerhardsson et al., Lam et al., and Wong et al. showed that CNS tumors were not significantly associated with lead exposure [36,39,43]. In contrast, those of Anttila et al., Barry et al., Cocco et al., Gwini et al., and Wijngaarden et al. showed that risk of CNS tumors was increased by lead exposure [14,34,42,44,47].

In these heterogeneous studies, some attempted to explain the results according to sex [15,38,40,45,46]. A few studies have reported sex-related differences in lead metabolism [48,49]. In studies showing results according to sex, most did not show a significant difference, which is consistent with our result.

In addition, studies of gene-environment interactions are getting more attention than in the past. The G177C polymorphism (rs1800435) of the d-amino-levulinic acid dehydratase (ALAD) gene has been reported as an effect modifier of lead exposure and was assessed in a study [11]. However, the study provided only preliminary evidence, so further studies should be performed.

### 4.2. Mechanism

Recent studies suggest that lead can cross the blood-brain barrier and concentrate in the brain parenchyma due to its ability to replace calcium ions [50]. Historically, neurotoxic effects of lead have been documented, especially during development in young children. In adults, several studies suggest that lead exposure is associated with neurodegenerative disease, including Alzheimer’s disease and Parkinson disease, by epigenetic modification, inhibition of N-methyl-D-aspartic acid receptor, and increased quinolinic acid production [9,51,52,53].

After absorption, lead is generally distributed to plasma, the nervous system, and soft tissues. In most studies, lead exposure resulted in micronucleus formation, chromosomal aberrations, and DNA damage in mammals [5].

The mechanism by which lead causes brain tumor is unclear. It has been proposed that lead can facilitate the process of carcinogenesis by inhibiting DNA synthesis and repair and by interacting with binding proteins, hindering tumor suppressor proteins. Lead may also affect carcinogenesis through mechanisms involving oxidative damage, induction of apoptosis, and altered signaling pathways [4,40,54,55].

### 4.3. Subgroup Analysis

In the subgroup analysis on exposure assessment method, significant differences were observed between the studies using blood lead level and the studies using job information. When we analyzed studies using blood lead level, significantly increased risks were identified in cases with all brain tumors as well as cases with malignant tumors only. When measuring lead exposure using job information, misclassification of subjects may occur, which can lead to underestimation of the risk. Moreover, there is potential bias from the patients’ subjective and selective recall of exposure. Therefore, we suggest that an objective measurement like blood lead level better reflects lead exposure, and this result also shows the association with brain tumors.

In the subgroup analysis of malignant brain tumors, the risk for brain cancer incidence was significantly higher than that for brain cancer mortality. The cause of death may be classified as a complication of brain cancer, not brain cancer itself. This misclassification can also underestimate the risks [56].

When performing subgroup analysis according to tumor subtypes, the risk of meningioma was slightly higher than that of glioma, although the difference between the two groups was not significant. The cause of this has not been studied yet. Although lead can cross the blood-brain barrier [50], this may be relatively difficult; therefore, lead might be more likely to reach the meninges than the brain parenchyma. Some previous studies about meningioma showed a significantly increased risk of meningioma [40,45], while another showed a non-significant increased risk [42]. Further research according to tumor subtypes is needed.

### 4.4. Strength and Limitation of This Study

We conducted subgroup analyses according to various factors, including research design, exposure assessment method, and outcome. Moreover, by performing statistical tests of publication bias, we determined that there is no evidence of publication bias in this study, and sensitivity analysis did not modify the results. Our study also has limitations. The included studies were heterogeneous in their study design, measurements of exposure, and tumor outcomes. Nevertheless, we sought to identify differences through subgroup analysis according to tumor type, study design, measurements of exposure, and tumor outcome to overcome this limitation. The issues of including gray literature and non-English publications are other possible limitations in the literature search.

## 5. Conclusions

In conclusion, this meta-analysis provides suggestive evidence for an association between lead compound exposure and brain tumor. We should pay attention to the risk of brain tumor associated with occupational lead exposure. Therefore, appropriate preventive strategies are needed for workers exposed to lead. Future studies evaluating the possible mechanisms of developing brain tumors and evaluating the risk according to brain tumor subtypes will be needed.

## Figures and Tables

**Table 1:** Characteristics of included studies.

Reference.	Country	Study Design	Study Period	Exposure Assessment	Reference Category	Sex	Tumor Type	Outcome	Study Quality	N (Ca/Co)
Gerhardsson et al. (1995) [43]	Sweden	Cohort	1969–1989	Blood lead level	External. (Swedish population)	M	CNS tumor (malignant)	Incidence	Med	1/664
Anttila et al. (1996) [14]	Finland	Case-control	1973–1990	Blood lead level	Internal. (lowest)	Both	CNS tumor (malignant)	Incidence	Med	26/20,741
							Glioma			16/20,741
Lundstrom et al. (1997) [37]	Sweden	Cohort	1958–1987	Job information	External. (Swedish population)	M	CNS tumor (malignant)	Incidence	Low	6/3979
Cocco et al. (1999) [42]	USA	Case-control	1984–1992	Job information	Internal. (unexposed)	F	Brain tumor (malignant)	Mortality	Low	12,980/51,920
							Meningioma (malignant)			161/51,920
Hu et al. (1999) [45]	China	Case-control	1989–1996	Job information	Internal. (unexposed)	M	Meningioma	Incidence	High	70/140
						F				113/226
Wong et al. (2000) [36]	USA	Cohort	1947–1995	Job information (Battery workers)	External. (American population)	M	CNS tumor	Mortality	High	10/4518
				Job information (Smelter workers)		M				5/2300
Wesseling et al. (2002) [35]	Finland	Cohort	1971–1995	Job information	External. (Finnish population)	F	CNS tumor	Incidence	High	693/413,877
van Wijngaarden et al. (2006) [34]	USA	Cohort	1979–1989	Job information	Internal. (unexposed)	Both	Brain tumor (malignant)	Mortality	Low	59,352/317,968
Lam et al. (2007) [39]	USA	Cohort	1985–2001	Job information	External. (American population)	M	Brain tumor (malignant)	Incidence	Med	2/3192
Prochazka et al. (2010) [38]	Sweden	Case-control	1987–1999	Job information	Internal. (unexposed)	Both	Acoustic neuroma (malignant)	Incidence	Low	793/101,762
						M				391/58,956
						F				402/42,806
Bhatti et al. (2011) [11]	USA	Case-control	1994–1998	Job information	Internal. (unexposed)	Both	Glioma	Incidence	High	355/505
							Meningioma			151/505
Gwini et al. (2012) [44]	Australia	Cohort	1983–2005	Job information	External. (Australian population)	M	CNS tumor (malignant)	Incidence	High	6/4114
Ilychova et al. (2012) [46]	Russia	Cohort	1979–2003	Job information	External. (Russian population)	M	CNS tumor (malignant)	Mortality	High	3/1423
						F				3/3102
Liao et al. (2016) [40]	China	Cohort	2000–2011	Job information	Internal. (unexposed)	M	Brain tumor (malignant)	Incidence	Low	35/61,466
			2004–2011	Job information		F	Brain tumor (malignant)	Incidence		42/73,363
						F	Meningioma			47/73,363
Sadetzk et al. (2016) [15]	7 countries	Case-control	2000–2004	Job information	Internal. (unexposed)	Both	Meningioma	Incidence	Low	1,906/5565
						M				507/2484
						F				1,399/3081
Parent et al. (2017) [41]	7 countries	Case-control	2000–2004	Job information	Internal. (unexposed)	Both	Glioma	Incidence	Low	1,800/5160
Barry et al. (2019) [47]	USA	Cohort	1987–2012	Blood lead level	Internal. (lowest)	M	CNS tumor (malignant)	Mortality	Low	56/58,368
Steenland et al. (2019) [10]	Finland	Cohort	1977–2013	Blood lead level	Internal. (lowest)	Both	CNS tumor	Incidence	Low	137/20,752
							CNS tumor (malignant)			68/20,752
							Glioma			55/20,752
	England	Cohort	1976–2011	Blood lead level	Internal. (lowest)	Both	CNS tumor		Low	24/9122
							CNS tumor (malignant)			17/9122
							Glioma			18/9122

Note: CNS, central nervous system; N, number of participants. Ca: cases/Co: controls or cohort.

**Table 2:** Results of subgroup analysis of all brain tumors (both benign and malignant).

Subgroup	No. of Studies	Pooled OR (95% CI)	Heterogeneity	
**Research design**			***I*^2^**, **%**	***p*-Value**
Case-control study	9	1.02 (0.82–1.27)	71	<0.01
Cohort study	15	1.27 (1.05–1.55)	0	0.8
Subgroup difference: *p* = 0.1319				
**Exposure assessment method**				
Blood lead level	6	1.67 (1.12–2.49)	0	0.96
Job information	18	1.04 (0.89–1.23)	52	<0.01
Subgroup difference: *p* = 0.0350				
**Tumor outcome**				
Brain tumor incidence	17	1.18 (0.94–1.49)	53	<0.01
Brain tumor mortality	7	1.1 (1.00–1.20)	0	0.81
Subgroup difference: *p* = 0.5639				
**Reference category**				
External reference	9	1.09 (0.83–1.42)	0	0.93
Internal reference	15	1.15 (0.95–1.40)	64	<0.01
Subgroup difference: *p* = 0.7157				

**Table 3:** Results of subgroup analyses of malignant brain tumors.

Subgroup	No. of Studies	Pooled OR (95% CI)	Heterogeneity	
**Research design**			***I*^2^, %**	***p*-Value**
Case-control study	3	1.10 (1.00–1.21)	0	0.4
Cohort study	15	1.28 (1.05–1.56)	0	0.74
Subgroup difference: *p* = 0.1790				
**Exposure assessment method**				
Blood lead (Pb) level	6	1.76 (1.16–2.69)	0	0.94
Job information	11	1.11 (1.02–1.21)	0	0.62
Subgroup difference: *p* = 0.0361				
**Cancer outcome**				
Brain cancer incidence	10	1.42 (1.09–1.85)	0	0.89
Brain cancer mortality	7	1.10 (1.01–1.21)	6	0.38
Subgroup difference: *p* = 0.0381				
**Reference category**				
External reference	9	1.09 (0.83–1.42)	0	0.93
Internal reference	9	1.14 (1.04–1.25)	0	0.67
Subgroup difference: *p* = 0.2594				

## Supplementary Materials

The following are available online at https://www.mdpi.com/1660-4601/17/11/3975/s1. Figure S1: Forest plot of the studies about the association between occupational lead exposure and all brain tumor after excluding studies with high risk of bias. Figure S2: Forest plot of the studies about the association between occupational lead exposure and all brain tumor after excluding studies with medium and high risk of bias. Figure S3: Funnel plot for the studies evaluating the association between occupational lead exposure and all brain tumors, before and after imputing missing studies with trim and fill method. Figure S4: Forest plot of the studies about the association between occupational lead exposure and malignant brain tumor after excluding studies with high risk of bias. Figure S5: Forest plot of the studies about the association between occupational lead exposure and malignant brain tumor after excluding studies with medium and high risk of bias. Figure S6: Funnel plot for the studies evaluating the association between occupational lead exposure and malignant brain tumors. Table S1: Subgroup analyses according to the tumor subtypes and sex.
