# Supplementary material for: Occupational Lead Exposure and Brain Tumors: Systematic Review and Meta-Analysis
Source: Int J Environ Res Public Health. 2020 Jun 3;17(11):3975. doi: 10.3390/ijerph17113975 (PMC7312535; doi:10.3390/ijerph17113975)
Supplement: Supplementary file 1 [file ijerph-17-03975-s001.pdf]

Supplementary Material 1. Methods: Search strategies for PubMed, EMBASE, Cochrane database

PubMed search details: 9,795 results

#1. "Occupations"[Mesh] OR "Occupations"[TW] OR "Occupation"[TW] OR "Vocations"[TW] OR "Vocation"[TW] OR "Occupational Exposure"[Mesh] OR "Occupational Exposure"[TW] OR "Exposure, Occupational"[TW] OR "Exposures, Occupational"[TW] OR "Occupational Exposures"[TW] OR "Occupational Diseases"[Mesh] OR "Occupational Diseases"[TW] OR "Disease, Occupational"[TW] OR "Occupational Disease"[TW] OR "Occupational Illnesses"[TW] OR "Illnesse, Occupational"[TW] OR "Illnesses, Occupational"[TW] OR "Occupational Illnesse"[TW] OR "Diseases, Occupational"[TW] OR "Work"[Mesh] OR "Work"[TW] OR "job"[TW] OR "worker"[TW] OR "workers"[TW] OR "occupational population"[TW] OR "Workplace"[Mesh] OR "Workplace"[TW] OR "Workplaces"[TW] OR "Work Location"[TW] OR "Location, Work"[TW] OR "Locations, Work"[TW] OR "Work Locations"[TW] OR "Work-Site"[TW] OR "Work Site"[TW] OR "Work-Sites"[TW] OR "Work Place"[TW] OR "Place, Work"[TW] OR "Places, Work"[TW] OR "Work Places"[TW] OR "Job Site"[TW] OR "Job Sites"[TW] OR "Site, Job"[TW] OR "Sites, Job"[TW] OR "Worksite"[TW] OR "Worksites"[TW] : 1,343,861 reults

#2: #1 AND "Lead"[Mesh] OR "Lead"[TW] OR "lead exposure"[TW] OR "Lead Poisoning"[Mesh] OR "Lead Poisoning"[TW] OR "Poisoning, Lead"[TW] OR "Lead Poisonings"[TW] OR "Poisonings, Lead"[TW] OR "Metals, Heavy"[Mesh] OR "Metals, Heavy"[TW] OR "Heavy Metals"[TW] OR "metal"[TW] OR "Heavy Metal Poisoning"[Mesh] OR "Heavy Metal Poisoning"[TW] OR "Heavy Metal Poisonings"[TW] OR "Metal Poisoning, Heavy"[TW] OR "Metal Poisonings, Heavy"[TW] OR "Poisoning, Heavy Metal"[TW] OR "Poisonings, Heavy Metal"[TW]: 96,290 results

#3: "Brain Neoplasms"[Mesh] OR "Brain Neoplasms"[TW] OR "Neoplasms, Brain"[TW] OR "Brain Neoplasm"[TW] OR "Neoplasm, Brain"[TW] OR "Brain Tumors"[TW] OR "Brain Tumor"[TW] OR "Tumor, Brain"[TW] OR "Tumors, Brain"[TW] OR "Brain Cancer"[TW] OR "Brain Cancers"[TW] OR "Cancer, Brain"[TW] OR "Cancers, Brain"[TW] OR "Malignant Neoplasms, Brain"[TW] OR "Brain Malignant Neoplasm"[TW] OR "Brain Malignant Neoplasms"[TW] OR "Malignant Neoplasm, Brain"[TW] OR "Cancer of Brain"[TW] OR "Cancer of the Brain"[TW] OR "Neoplasms, Brain, Malignant"[TW] OR "Brain Neoplasms, Malignant"[TW] OR "Brain Neoplasm, Malignant"[TW] OR "Malignant Brain Neoplasm"[TW] OR "Malignant Brain Neoplasms"[TW] OR "Neoplasms, Intracranial"[TW] OR "Intracranial Neoplasm"[TW] OR "Neoplasm, Intracranial"[TW] OR "Intracranial Neoplasms"[TW] OR "Brain Tumor, Recurrent"[TW] OR "Brain Tumors, Recurrent"[TW] OR "Recurrent Brain Tumor"[TW] OR "Recurrent Brain Tumors"[TW] OR "Malignant Primary Brain Tumors"[TW] OR "Primary Malignant Brain Tumors"[TW] OR "Malignant Primary Brain Neoplasms"[TW] OR "Primary Malignant Brain Neoplasms"[TW] OR "Brain Neoplasms, Malignant, Primary"[TW] OR "Brain Neoplasms, Primary Malignant"[TW] OR "Benign Neoplasms, Brain"[TW] OR "Benign Neoplasm, Brain"[TW] OR "Brain Benign Neoplasm"[TW] OR "Brain Benign Neoplasms"[TW] OR "Neoplasms, Brain, Benign"[TW] OR "Brain Neoplasms, Benign"[TW] OR "Benign Brain Neoplasm"[TW] OR "Benign Brain Neoplasms"[TW] OR "Brain Neoplasm, Benign"[TW] OR "Brain Tumor, Primary"[TW] OR "Brain Tumors, Primary"[TW] OR "Primary Brain Tumor"[TW] OR "Primary Brain Tumors"[TW] OR "Neoplasms, Brain, Primary"[TW] OR "Brain Neoplasm, Primary"[TW] OR "Brain Neoplasms, Primary"[TW] OR "Primary Brain Neoplasm"[TW] OR "Primary Brain Neoplasms"[TW] OR "brain malignant"[TW] OR "brain carcinoma"[TW] OR "malignant"[TW] OR "Glioma"[Mesh] OR "Glioma"[TW] OR "Gliomas"[TW] OR "Glial Cell Tumors"[TW] OR "Glial Cell Tumor"[TW] OR "Tumor, Glial Cell"[TW] OR "Tumors, Glial Cell"[TW] OR "Mixed Glioma"[TW] OR "Glioma, Mixed"[TW] OR "Gliomas, Mixed"[TW] OR "Mixed Gliomas"[TW] OR "Malignant Glioma"[TW] OR "Glioma, Malignant"[TW] OR "Gliomas, Malignant"[TW] OR "Malignant Gliomas"[TW] OR "Meningioma"[Mesh] OR "Meningioma"[TW] OR "Meningiomas"[TW] OR "Xanthomatous Meningioma"[TW] OR "Meningioma, Xanthomatous"[TW] OR "Meningiomas, Xanthomatous"[TW] OR "Xanthomatous Meningiomas"[TW] OR "Angioblastic Meningioma"[TW] OR "Angioblastic Meningiomas"[TW] OR "Meningioma, Angioblastic"[TW] OR

"Meningiomas, Angioblastic"[TW] OR "Angiomatous Meningioma"[TW] OR "Angiomatous Meningiomas"[TW] OR "Meningioma, Angiomatous"[TW] OR "Meningiomas, Angiomatous"[TW] OR "Clear Cell Meningioma"[TW] OR "Clear Cell Meningiomas"[TW] OR "Meningioma, Clear Cell"[TW] OR "Meningiomas, Clear Cell"[TW] OR "Fibrous Meningioma"[TW] OR "Fibrous Meningiomas"[TW] OR "Meningioma, Fibrous"[TW] OR "Meningiomas, Fibrous"[TW] OR "Hemangioblastic Meningioma"[TW] OR "Hemangioblastic Meningiomas"[TW] OR "Meningioma, Hemangioblastic"[TW] OR "Meningiomas, Hemangioblastic"[TW] OR "Hemangiopericytic Meningioma"[TW] OR "Hemangiopericytic Meningiomas"[TW] OR "Meningioma, Hemangiopericytic"[TW] OR "Meningiomas, Hemangiopericytic"[TW] OR "Intracranial Meningioma"[TW] OR "Intracranial Meningiomas"[TW] OR "Meningioma, Intracranial"[TW] OR "Meningiomas, Intracranial"[TW] OR "Intraorbital Meningioma"[TW] OR "Intraorbital Meningiomas"[TW] OR "Meningioma, Intraorbital"[TW] OR "Meningiomas, Intraorbital"[TW] OR "Intraventricular Meningioma"[TW] OR "Intraventricular Meningiomas"[TW] OR "Meningioma, Intraventricular"[TW] OR "Meningiomas, Intraventricular"[TW] OR "Malignant Meningioma"[TW] OR "Malignant Meningiomas"[TW] OR "Meningioma, Malignant"[TW] OR "Meningiomas, Malignant"[TW] OR "Meningiomas, Multiple"[TW] OR "Meningioma, Multiple"[TW] OR "Multiple Meningioma"[TW] OR "Multiple Meningiomas"[TW] OR "Meningiomatosis"[TW] OR "Meningiomatoses"[TW] OR "Meningotheliomatous Meningioma"[TW] OR "Meningioma, Meningotheliomatous"[TW] OR "Meningiomas, Meningotheliomatous"[TW] OR "Meningotheliomatous Meningiomas"[TW] OR "Microcystic Meningioma"[TW] OR "Meningioma, Microcystic"[TW] OR "Meningiomas, Microcystic"[TW] OR "Microcystic Meningiomas"[TW] OR "Olfactory Groove Meningioma"[TW] OR "Groove Meningiomas, Olfactory"[TW] OR "Meningioma, Olfactory Groove"[TW] OR "Meningiomas, Olfactory Groove"[TW] OR "Olfactory Groove Meningiomas"[TW] OR "Papillary Meningioma"[TW] OR "Meningioma, Papillary"[TW] OR "Meningiomas, Papillary"[TW] OR "Papillary Meningiomas"[TW] OR "Parasagittal Meningioma"[TW] OR "Meningioma, Parasagittal"[TW] OR "Meningiomas, Parasagittal"[TW] OR "Parasagittal Meningiomas"[TW] OR "Posterior Fossa Meningioma"[TW] OR "Meningioma, Posterior Fossa"[TW] OR "Meningiomas, Posterior Fossa"[TW] OR "Posterior Fossa Meningiomas"[TW] OR "Psammomatous Meningioma"[TW] OR "Meningioma, Psammomatous"[TW] OR "Meningiomas, Psammomatous"[TW] OR "Psammomatous Meningiomas"[TW] OR "Secretory Meningioma"[TW] OR "Meningioma, Secretory"[TW] OR "Meningiomas, Secretory"[TW] OR "Secretory Meningiomas"[TW] OR "Sphenoid Wing Meningioma"[TW] OR "Meningioma, Sphenoid Wing"[TW] OR "Meningiomas, Sphenoid Wing"[TW] OR "Sphenoid Wing Meningiomas"[TW] OR "Wing Meningioma, Sphenoid"[TW] OR "Wing Meningiomas, Sphenoid"[TW] OR "Spinal Meningioma"[TW] OR "Meningioma, Spinal"[TW] OR "Meningiomas, Spinal"[TW] OR "Spinal Meningiomas"[TW] OR "Transitional Meningioma"[TW] OR "Meningioma, Transitional"[TW] OR "Meningiomas, Transitional"[TW] OR "Transitional Meningiomas"[TW] OR "Benign Meningioma"[TW] OR "Benign Meningiomas"[TW] OR "Meningioma, Benign"[TW] OR "Meningiomas, Benign"[TW] OR "Cerebral Convexity Meningioma"[TW] OR "Cerebral Convexity Meningiomas"[TW] OR "Convexity Meningioma, Cerebral"[TW] OR "Convexity Meningiomas, Cerebral"[TW] OR "Meningioma, Cerebral Convexity"[TW] OR "Meningiomas, Cerebral Convexity"[TW] OR "Carcinoma"[Mesh] OR "Carcinoma"[TW] OR "Carcinomas"[TW] OR "Epithelial Neoplasms, Malignant"[TW] OR "Malignant Epithelial Neoplasms"[TW] OR "Epithelial Neoplasm, Malignant"[TW] OR "Malignant Epithelial Neoplasm"[TW] OR "Neoplasm, Malignant Epithelial"[TW] OR "Neoplasms, Malignant Epithelial"[TW] OR "Epithelial Tumors, Malignant"[TW] OR "Epithelial Tumor, Malignant"[TW] OR "Malignant Epithelial Tumor"[TW] OR "Malignant Epithelial Tumors"[TW] OR "Tumor, Malignant Epithelial"[TW] OR "Epithelioma"[TW] OR "Epitheliomas"[TW] OR "Carcinoma, Undifferentiated"[TW] OR "Undifferentiated Carcinoma"[TW] OR "Undifferentiated Carcinomas"[TW] OR "Carcinoma, Anaplastic"[TW] OR "Anaplastic Carcinoma"[TW] OR "Anaplastic Carcinomas"[TW] OR "Carcinoma, Spindle-Cell"[TW] OR "Carcinoma, Spindle Cell"[TW] OR "Spindle-Cell Carcinoma"[TW] OR "Spindle-Cell Carcinomas"[TW] OR "Carcinomatosis"[TW] OR "Carcinomatoses"[TW] OR "Neoplasms"[Mesh] OR "Neoplasms"[TW] OR "Neoplasia"[TW] OR "Neoplasias"[TW] OR "Neoplasm"[TW] OR

"Tumors"[TW] OR "Tumor"[TW] OR "Cancer"[TW] OR "Cancers"[TW] OR "Malignancy"[TW] OR "Malignancies"[TW] OR "Malignant Neoplasms"[TW] OR "Malignant Neoplasm"[TW] OR "Neoplasm, Malignant"[TW] OR "Neoplasms, Malignant"[TW] OR "Benign Neoplasms"[TW] OR "Neoplasms, Benign"[TW] OR "Benign Neoplasm"[TW] OR "Neoplasm, Benign"[TW]: 4,284,259

#4. #2 AND #3: 9,795 reults

EMBASE: 13,841 results

#1.'occupation'/exp OR ('occupations':ti,ab,kw,de OR 'occupation':ti,ab,kw,de OR 'vocations':ti,ab,kw,de OR 'vocation':ti,ab,kw,de) OR 'occupational exposure'/exp OR ('occupational exposure':ti,ab,kw,de OR 'exposure, occupational':ti,ab,kw,de OR 'exposures, occupational':ti,ab,kw,de OR 'occupational exposures':ti,ab,kw,de) OR 'occupational disease'/exp OR ('occupational diseases':ti,ab,kw,de OR 'disease, occupational':ti,ab,kw,de OR 'occupational disease':ti,ab,kw,de OR 'occupational illnesses':ti,ab,kw,de OR 'illnesse, occupational':ti,ab,kw,de OR 'illnesses, occupational':ti,ab,kw,de OR 'occupational illnesse':ti,ab,kw,de OR 'diseases, occupational':ti,ab,kw,de) OR 'work'/exp OR ('work':ti,ab,kw,de OR 'job':ti,ab,kw,de OR 'worker':ti,ab,kw,de OR 'workers':ti,ab,kw,de OR 'occupational population':ti,ab,kw,de) OR 'workplace'/exp OR ('workplace':ti,ab,kw,de OR 'workplaces':ti,ab,kw,de OR 'work location':ti,ab,kw,de OR 'location, work':ti,ab,kw,de OR 'locations, work':ti,ab,kw,de OR 'work locations':ti,ab,kw,de OR 'work-site':ti,ab,kw,de OR 'work site':ti,ab,kw,de OR 'work-sites':ti,ab,kw,de OR 'work place':ti,ab,kw,de OR 'place, work':ti,ab,kw,de OR 'places, work':ti,ab,kw,de OR 'work places':ti,ab,kw,de OR 'job site':ti,ab,kw,de OR 'job sites':ti,ab,kw,de OR 'site, job':ti,ab,kw,de OR 'sites, job':ti,ab,kw,de OR 'worksite':ti,ab,kw,de OR 'worksites':ti,ab,kw,de): 2,021,060 results

#2. AND 'lead'/exp OR ('lead':ti,ab,kw,de OR 'lead exposure':ti,ab,kw,de) OR 'lead poisoning'/exp OR ('lead poisoning':ti,ab,kw,de OR 'poisoning, lead':ti,ab,kw,de OR 'lead poisonings':ti,ab,kw,de OR 'poisonings, lead':ti,ab,kw,de) OR 'heavy metal'/exp OR ('metals, heavy':ti,ab,kw,de OR 'heavy metals':ti,ab,kw,de OR 'metal':ti,ab,kw,de) OR 'heavy metal poisoning'/exp OR ('heavy metal poisoning':ti,ab,kw,de OR 'heavy metal poisonings':ti,ab,kw,de OR 'metal poisoning, heavy':ti,ab,kw,de OR 'metal poisonings, heavy':ti,ab,kw,de OR 'poisoning, heavy metal':ti,ab,kw,de OR 'poisonings, heavy metal':ti,ab,kw,de): 109,204

#3. 'brain tumor'/exp OR ('brain neoplasms':ti,ab,kw,de OR 'neoplasms, brain':ti,ab,kw,de OR 'brain neoplasm':ti,ab,kw,de OR 'neoplasm, brain':ti,ab,kw,de OR 'brain tumors':ti,ab,kw,de OR 'brain tumor':ti,ab,kw,de OR 'tumor, brain':ti,ab,kw,de OR 'tumors, brain':ti,ab,kw,de OR 'brain cancer':ti,ab,kw,de OR 'brain cancers':ti,ab,kw,de OR 'cancer, brain':ti,ab,kw,de OR 'cancers, brain':ti,ab,kw,de OR 'malignant neoplasms, brain':ti,ab,kw,de OR 'brain malignant neoplasm':ti,ab,kw,de OR 'brain malignant neoplasms':ti,ab,kw,de OR 'malignant neoplasm, brain':ti,ab,kw,de OR 'cancer of brain':ti,ab,kw,de OR 'cancer of the brain':ti,ab,kw,de OR 'neoplasms, brain, malignant':ti,ab,kw,de OR 'brain neoplasms, malignant':ti,ab,kw,de OR 'brain neoplasm, malignant':ti,ab,kw,de OR 'malignant brain neoplasm':ti,ab,kw,de OR 'malignant brain neoplasms':ti,ab,kw,de OR 'neoplasms, intracranial':ti,ab,kw,de OR 'intracranial neoplasm':ti,ab,kw,de OR 'neoplasm, intracranial':ti,ab,kw,de OR 'intracranial neoplasms':ti,ab,kw,de OR 'brain tumor, recurrent':ti,ab,kw,de OR 'brain tumors, recurrent':ti,ab,kw,de OR 'recurrent brain tumor':ti,ab,kw,de OR 'recurrent brain tumors':ti,ab,kw,de OR 'malignant primary brain tumors':ti,ab,kw,de OR 'primary malignant brain tumors':ti,ab,kw,de OR 'malignant primary brain neoplasms':ti,ab,kw,de OR 'primary malignant brain neoplasms':ti,ab,kw,de OR 'brain neoplasms, malignant, primary':ti,ab,kw,de OR 'brain neoplasms, primary malignant':ti,ab,kw,de OR 'benign neoplasms, brain':ti,ab,kw,de OR 'benign neoplasm, brain':ti,ab,kw,de OR 'brain benign neoplasm':ti,ab,kw,de OR 'brain benign neoplasms':ti,ab,kw,de OR 'neoplasms, brain, benign':ti,ab,kw,de OR 'brain neoplasms, benign':ti,ab,kw,de OR 'benign brain neoplasm':ti,ab,kw,de OR 'benign brain neoplasms':ti,ab,kw,de OR 'brain neoplasm,

benign':ti,ab,kw,de OR 'brain tumor, primary':ti,ab,kw,de OR 'brain tumors, primary':ti,ab,kw,de OR 'primary brain tumor':ti,ab,kw,de OR 'primary brain tumors':ti,ab,kw,de OR 'neoplasms, brain, primary':ti,ab,kw,de OR 'brain neoplasm, primary':ti,ab,kw,de OR 'brain neoplasms, primary':ti,ab,kw,de OR 'primary brain neoplasm':ti,ab,kw,de OR 'primary brain neoplasms':ti,ab,kw,de OR 'brain malignant':ti,ab,kw,de OR 'brain carcinoma':ti,ab,kw,de OR 'malignant':ti,ab,kw,de) OR 'glioma'/exp OR ('glioma':ti,ab,kw,de OR 'gliomas':ti,ab,kw,de OR 'glial cell tumors':ti,ab,kw,de OR 'glial cell tumor':ti,ab,kw,de OR 'tumor, glial cell':ti,ab,kw,de OR 'tumors, glial cell':ti,ab,kw,de OR 'mixed glioma':ti,ab,kw,de OR 'glioma, mixed':ti,ab,kw,de OR 'gliomas, mixed':ti,ab,kw,de OR 'mixed gliomas':ti,ab,kw,de OR 'malignant glioma':ti,ab,kw,de OR 'glioma, malignant':ti,ab,kw,de OR 'gliomas, malignant':ti,ab,kw,de OR 'malignant gliomas':ti,ab,kw,de) OR 'meningioma'/exp OR ('meningioma':ti,ab,kw,de OR 'meningiomas':ti,ab,kw,de OR 'xanthomatous meningioma':ti,ab,kw,de OR 'meningioma, xanthomatous':ti,ab,kw,de OR 'meningiomas, xanthomatous':ti,ab,kw,de OR 'xanthomatous meningiomas':ti,ab,kw,de OR 'angioblastic meningioma':ti,ab,kw,de OR 'angioblastic meningiomas':ti,ab,kw,de OR 'meningioma, angioblastic':ti,ab,kw,de OR 'meningiomas, angioblastic':ti,ab,kw,de OR 'angiomatous meningioma':ti,ab,kw,de OR 'angiomatous meningiomas':ti,ab,kw,de OR 'meningioma, angiomatous':ti,ab,kw,de OR 'meningiomas, angiomatous':ti,ab,kw,de OR 'clear cell meningioma':ti,ab,kw,de OR 'clear cell meningiomas':ti,ab,kw,de OR 'meningioma, clear cell':ti,ab,kw,de OR 'meningiomas, clear cell':ti,ab,kw,de OR 'fibrous meningioma':ti,ab,kw,de OR 'fibrous meningiomas':ti,ab,kw,de OR 'meningioma, fibrous':ti,ab,kw,de OR 'meningiomas, fibrous':ti,ab,kw,de OR 'hemangioblastic meningioma':ti,ab,kw,de OR 'hemangioblastic meningiomas':ti,ab,kw,de OR 'meningioma, hemangioblastic':ti,ab,kw,de OR 'meningiomas, hemangioblastic':ti,ab,kw,de OR 'hemangiopericytic meningioma':ti,ab,kw,de OR 'hemangiopericytic meningiomas':ti,ab,kw,de OR 'meningioma, hemangiopericytic':ti,ab,kw,de OR 'meningiomas, hemangiopericytic':ti,ab,kw,de OR 'intracranial meningioma':ti,ab,kw,de OR 'intracranial meningiomas':ti,ab,kw,de OR 'meningioma, intracranial':ti,ab,kw,de OR 'meningiomas, intracranial':ti,ab,kw,de OR 'intraorbital meningioma':ti,ab,kw,de OR 'intraorbital meningiomas':ti,ab,kw,de OR 'meningioma, intraorbital':ti,ab,kw,de OR 'meningiomas, intraorbital':ti,ab,kw,de OR 'intraventricular meningioma':ti,ab,kw,de OR 'intraventricular meningiomas':ti,ab,kw,de OR 'meningioma, intraventricular':ti,ab,kw,de OR 'meningiomas, intraventricular':ti,ab,kw,de OR 'malignant meningioma':ti,ab,kw,de OR 'malignant meningiomas':ti,ab,kw,de OR 'meningioma, malignant':ti,ab,kw,de OR 'meningiomas, malignant':ti,ab,kw,de OR 'meningiomas, multiple':ti,ab,kw,de OR 'meningioma, multiple':ti,ab,kw,de OR 'multiple meningioma':ti,ab,kw,de OR 'multiple meningiomas':ti,ab,kw,de OR 'meningiomatosis':ti,ab,kw,de OR 'meningiomatoses':ti,ab,kw,de OR 'meningotheliomatous meningioma':ti,ab,kw,de OR 'meningioma, meningotheliomatous':ti,ab,kw,de OR 'meningiomas, meningotheliomatous':ti,ab,kw,de OR 'meningotheliomatous meningiomas':ti,ab,kw,de OR 'microcystic meningioma':ti,ab,kw,de OR 'meningioma, microcystic':ti,ab,kw,de OR 'meningiomas, microcystic':ti,ab,kw,de OR 'microcystic meningiomas':ti,ab,kw,de OR 'olfactory groove meningioma':ti,ab,kw,de OR 'groove meningiomas, olfactory':ti,ab,kw,de OR 'meningioma, olfactory groove':ti,ab,kw,de OR 'meningiomas, olfactory groove':ti,ab,kw,de OR 'olfactory groove meningiomas':ti,ab,kw,de OR 'papillary meningioma':ti,ab,kw,de OR 'meningioma, papillary':ti,ab,kw,de OR 'meningiomas, papillary':ti,ab,kw,de OR 'papillary meningiomas':ti,ab,kw,de OR 'parasagittal meningioma':ti,ab,kw,de OR 'meningioma, parasagittal':ti,ab,kw,de OR 'meningiomas, parasagittal':ti,ab,kw,de OR 'parasagittal meningiomas':ti,ab,kw,de OR 'posterior fossa meningioma':ti,ab,kw,de OR 'meningioma, posterior fossa':ti,ab,kw,de OR 'meningiomas, posterior fossa':ti,ab,kw,de OR 'posterior fossa meningiomas':ti,ab,kw,de OR 'psammomatous meningioma':ti,ab,kw,de OR 'meningioma, psammomatous':ti,ab,kw,de OR 'meningiomas, psammomatous':ti,ab,kw,de OR 'psammomatous meningiomas':ti,ab,kw,de OR 'secretory meningioma':ti,ab,kw,de OR 'meningioma, secretory':ti,ab,kw,de OR 'meningiomas, secretory':ti,ab,kw,de OR 'secretory meningiomas':ti,ab,kw,de OR 'sphenoid wing meningioma':ti,ab,kw,de OR 'meningioma, sphenoid wing':ti,ab,kw,de OR 'meningiomas, sphenoid wing':ti,ab,kw,de OR 'sphenoid wing

meningiomas':ti,ab,kw,de OR 'wing meningioma, sphenoid':ti,ab,kw,de OR 'wing meningiomas, sphenoid':ti,ab,kw,de OR 'spinal meningioma':ti,ab,kw,de OR 'meningioma, spinal':ti,ab,kw,de OR 'meningiomas, spinal':ti,ab,kw,de OR 'spinal meningiomas':ti,ab,kw,de OR 'transitional meningioma':ti,ab,kw,de OR 'meningioma, transitional':ti,ab,kw,de OR 'meningiomas, transitional':ti,ab,kw,de OR 'transitional meningiomas':ti,ab,kw,de OR 'benign meningioma':ti,ab,kw,de OR 'benign meningiomas':ti,ab,kw,de OR 'meningioma, benign':ti,ab,kw,de OR 'meningiomas, benign':ti,ab,kw,de OR 'cerebral convexity meningioma':ti,ab,kw,de OR 'cerebral convexity meningiomas':ti,ab,kw,de OR 'convexity meningioma, cerebral':ti,ab,kw,de OR 'convexity meningiomas, cerebral':ti,ab,kw,de OR 'meningioma, cerebral convexity':ti,ab,kw,de OR 'meningiomas, cerebral convexity':ti,ab,kw,de) OR 'carcinoma'/exp OR ('carcinoma':ti,ab,kw,de OR 'carcinomas':ti,ab,kw,de OR 'epithelial neoplasms, malignant':ti,ab,kw,de OR 'malignant epithelial neoplasms':ti,ab,kw,de OR 'epithelial neoplasm, malignant':ti,ab,kw,de OR 'malignant epithelial neoplasm':ti,ab,kw,de OR 'neoplasm, malignant epithelial':ti,ab,kw,de OR 'neoplasms, malignant epithelial':ti,ab,kw,de OR 'epithelial tumors, malignant':ti,ab,kw,de OR 'epithelial tumor, malignant':ti,ab,kw,de OR 'malignant epithelial tumor':ti,ab,kw,de OR 'malignant epithelial tumors':ti,ab,kw,de OR 'tumor, malignant epithelial':ti,ab,kw,de OR 'epithelioma':ti,ab,kw,de OR 'epitheliomas':ti,ab,kw,de OR 'carcinoma, undifferentiated':ti,ab,kw,de OR 'undifferentiated carcinoma':ti,ab,kw,de OR 'undifferentiated carcinomas':ti,ab,kw,de OR 'carcinoma, anaplastic':ti,ab,kw,de OR 'anaplastic carcinoma':ti,ab,kw,de OR 'anaplastic carcinomas':ti,ab,kw,de OR 'carcinoma, spindle-cell':ti,ab,kw,de OR 'carcinoma, spindle cell':ti,ab,kw,de OR 'spindle-cell carcinoma':ti,ab,kw,de OR 'spindle-cell carcinomas':ti,ab,kw,de OR 'carcinomatosis':ti,ab,kw,de OR 'carcinomatoses':ti,ab,kw,de) OR 'neoplasm'/exp OR ('neoplasms':ti,ab,kw,de OR 'neoplasia':ti,ab,kw,de OR 'neoplasias':ti,ab,kw,de OR 'neoplasm':ti,ab,kw,de OR 'tumors':ti,ab,kw,de OR 'tumor':ti,ab,kw,de OR 'cancer':ti,ab,kw,de OR 'cancers':ti,ab,kw,de OR 'malignancy':ti,ab,kw,de OR 'malignancies':ti,ab,kw,de OR 'malignant neoplasms':ti,ab,kw,de OR 'malignant neoplasm':ti,ab,kw,de OR 'neoplasm, malignant':ti,ab,kw,de OR 'neoplasms, malignant':ti,ab,kw,de OR 'benign neoplasms':ti,ab,kw,de OR 'neoplasms, benign':ti,ab,kw,de OR 'benign neoplasm':ti,ab,kw,de OR 'neoplasm, benign':ti,ab,kw,de): 5,905,609 results

#4: #2 AND #3: 13,841 results

Cochrane database:

#1. [mh "Occupations"] OR "Occupations":ti,ab,kw OR "Occupation":ti,ab,kw OR "Vocations":ti,ab,kw OR "Vocation":ti,ab,kw OR [mh "Occupational Exposure"] OR "Occupational Exposure":ti,ab,kw OR "Exposure, Occupational":ti,ab,kw OR "Exposures, Occupational":ti,ab,kw OR "Occupational Exposures":ti,ab,kw OR [mh "Occupational Diseases"] OR "Occupational Diseases":ti,ab,kw OR "Disease, Occupational":ti,ab,kw OR "Occupational Disease":ti,ab,kw OR "Occupational Illnesses":ti,ab,kw OR "Illnesse, Occupational":ti,ab,kw OR "Illnesses, Occupational":ti,ab,kw OR "Occupational Illnesse":ti,ab,kw OR "Diseases, Occupational":ti,ab,kw OR [mh "Work"] OR "Work":ti,ab,kw OR "job":ti,ab,kw OR "worker":ti,ab,kw OR "workers":ti,ab,kw OR "occupational population":ti,ab,kw OR [mh "Workplace"] OR "Workplace":ti,ab,kw OR "Workplaces":ti,ab,kw OR "Work Location":ti,ab,kw OR "Location, Work":ti,ab,kw OR "Locations, Work":ti,ab,kw OR "Work Locations":ti,ab,kw OR "Work-Site":ti,ab,kw OR "Work Site":ti,ab,kw OR "Work-Sites":ti,ab,kw OR "Work Place":ti,ab,kw OR "Place, Work":ti,ab,kw OR "Places, Work":ti,ab,kw OR "Work Places":ti,ab,kw OR "Job Site":ti,ab,kw OR "Job Sites":ti,ab,kw OR "Site, Job":ti,ab,kw OR "Sites, Job":ti,ab,kw OR "Worksite":ti,ab,kw OR "Worksites":ti,ab,kw : 51,934 results

#2. AND [mh "Lead"] OR "Lead":ti,ab,kw OR "lead exposure":ti,ab,kw OR [mh "Lead Poisoning"] OR "Lead Poisoning":ti,ab,kw OR "Poisoning, Lead":ti,ab,kw OR "Lead Poisonings":ti,ab,kw OR "Poisonings, Lead":ti,ab,kw OR [mh "Metals, Heavy"] OR "Metals, Heavy":ti,ab,kw OR "Heavy Metals":ti,ab,kw OR "metal":ti,ab,kw OR [mh "Heavy Metal Poisoning"] OR "Heavy Metal Poisoning":ti,ab,kw OR "Heavy Metal Poisonings":ti,ab,kw OR "Metal Poisoning, Heavy":ti,ab,kw OR "Metal Poisonings, Heavy":ti,ab,kw OR "Poisoning, Heavy Metal":ti,ab,kw OR "Poisonings,

Heavy Metal":ti,ab,kw : 2,647 results

#3. [mh "Brain Neoplasms"] OR "Brain Neoplasms":ti,ab,kw OR "Neoplasms, Brain":ti,ab,kw OR "Brain Neoplasm":ti,ab,kw OR "Neoplasm, Brain":ti,ab,kw OR "Brain Tumors":ti,ab,kw OR "Brain Tumor":ti,ab,kw OR "Tumor, Brain":ti,ab,kw OR "Tumors, Brain":ti,ab,kw OR "Brain Cancer":ti,ab,kw OR "Brain Cancers":ti,ab,kw OR "Cancer, Brain":ti,ab,kw OR "Cancers, Brain":ti,ab,kw OR "Malignant Neoplasms, Brain":ti,ab,kw OR "Brain Malignant Neoplasm":ti,ab,kw OR "Brain Malignant Neoplasms":ti,ab,kw OR "Malignant Neoplasm, Brain":ti,ab,kw OR "Cancer of Brain":ti,ab,kw OR "Cancer of the Brain":ti,ab,kw OR "Neoplasms, Brain, Malignant":ti,ab,kw OR "Brain Neoplasms, Malignant":ti,ab,kw OR "Brain Neoplasm, Malignant":ti,ab,kw OR "Malignant Brain Neoplasm":ti,ab,kw OR "Malignant Brain Neoplasms":ti,ab,kw OR "Neoplasms, Intracranial":ti,ab,kw OR "Intracranial Neoplasm":ti,ab,kw OR "Neoplasm, Intracranial":ti,ab,kw OR "Intracranial Neoplasms":ti,ab,kw OR "Brain Tumor, Recurrent":ti,ab,kw OR "Brain Tumors, Recurrent":ti,ab,kw OR "Recurrent Brain Tumor":ti,ab,kw OR "Recurrent Brain Tumors":ti,ab,kw OR "Malignant Primary Brain Tumors":ti,ab,kw OR "Primary Malignant Brain Tumors":ti,ab,kw OR "Malignant Primary Brain Neoplasms":ti,ab,kw OR "Primary Malignant Brain Neoplasms":ti,ab,kw OR "Brain Neoplasms, Malignant, Primary":ti,ab,kw OR "Brain Neoplasms, Primary Malignant":ti,ab,kw OR "Benign Neoplasms, Brain":ti,ab,kw OR "Benign Neoplasm, Brain":ti,ab,kw OR "Brain Benign Neoplasm":ti,ab,kw OR "Brain Benign Neoplasms":ti,ab,kw OR "Neoplasms, Brain, Benign":ti,ab,kw OR "Brain Neoplasms, Benign":ti,ab,kw OR "Benign Brain Neoplasm":ti,ab,kw OR "Benign Brain Neoplasms":ti,ab,kw OR "Brain Neoplasm, Benign":ti,ab,kw OR "Brain Tumor, Primary":ti,ab,kw OR "Brain Tumors, Primary":ti,ab,kw OR "Primary Brain Tumor":ti,ab,kw OR "Primary Brain Tumors":ti,ab,kw OR "Neoplasms, Brain, Primary":ti,ab,kw OR "Brain Neoplasm, Primary":ti,ab,kw OR "Brain Neoplasms, Primary":ti,ab,kw OR "Primary Brain Neoplasm":ti,ab,kw OR "Primary Brain Neoplasms":ti,ab,kw OR "brain malignant":ti,ab,kw OR "brain carcinoma":ti,ab,kw OR "malignant":ti,ab,kw OR [mh "Glioma"] OR "Glioma":ti,ab,kw OR "Gliomas":ti,ab,kw OR "Glial Cell Tumors":ti,ab,kw OR "Glial Cell Tumor":ti,ab,kw OR "Tumor, Glial Cell":ti,ab,kw OR "Tumors, Glial Cell":ti,ab,kw OR "Mixed Glioma":ti,ab,kw OR "Glioma, Mixed":ti,ab,kw OR "Gliomas, Mixed":ti,ab,kw OR "Mixed Gliomas":ti,ab,kw OR "Malignant Glioma":ti,ab,kw OR "Glioma, Malignant":ti,ab,kw OR "Gliomas, Malignant":ti,ab,kw OR "Malignant Gliomas":ti,ab,kw OR [mh "Meningioma"] OR "Meningioma":ti,ab,kw OR "Meningiomas":ti,ab,kw OR "Xanthomatous Meningioma":ti,ab,kw OR "Meningioma, Xanthomatous":ti,ab,kw OR "Meningiomas, Xanthomatous":ti,ab,kw OR "Xanthomatous Meningiomas":ti,ab,kw OR "Angioblastic Meningioma":ti,ab,kw OR "Angioblastic Meningiomas":ti,ab,kw OR "Meningioma, Angioblastic":ti,ab,kw OR "Meningiomas, Angioblastic":ti,ab,kw OR "Angiomatous Meningioma":ti,ab,kw OR "Angiomatous Meningiomas":ti,ab,kw OR "Meningioma, Angiomatous":ti,ab,kw OR "Meningiomas, Angiomatous":ti,ab,kw OR "Clear Cell Meningioma":ti,ab,kw OR "Clear Cell Meningiomas":ti,ab,kw OR "Meningioma, Clear Cell":ti,ab,kw OR "Meningiomas, Clear Cell":ti,ab,kw OR "Fibrous Meningioma":ti,ab,kw OR "Fibrous Meningiomas":ti,ab,kw OR "Meningioma, Fibrous":ti,ab,kw OR "Meningiomas, Fibrous":ti,ab,kw OR "Hemangioblastic Meningioma":ti,ab,kw OR "Hemangioblastic Meningiomas":ti,ab,kw OR "Meningioma, Hemangioblastic":ti,ab,kw OR "Meningiomas, Hemangioblastic":ti,ab,kw OR "Hemangiopericytic Meningioma":ti,ab,kw OR "Hemangiopericytic Meningiomas":ti,ab,kw OR "Meningioma, Hemangiopericytic":ti,ab,kw OR "Meningiomas, Hemangiopericytic":ti,ab,kw OR "Intracranial Meningioma":ti,ab,kw OR "Intracranial Meningiomas":ti,ab,kw OR "Meningioma, Intracranial":ti,ab,kw OR "Meningiomas, Intracranial":ti,ab,kw OR "Intraorbital Meningioma":ti,ab,kw OR "Intraorbital Meningiomas":ti,ab,kw OR "Meningioma, Intraorbital":ti,ab,kw OR "Meningiomas, Intraorbital":ti,ab,kw OR "Intraventricular Meningioma":ti,ab,kw OR "Intraventricular Meningiomas":ti,ab,kw OR "Meningioma, Intraventricular":ti,ab,kw OR "Meningiomas, Intraventricular":ti,ab,kw OR "Malignant Meningioma":ti,ab,kw OR "Malignant Meningiomas":ti,ab,kw OR "Meningioma, Malignant":ti,ab,kw OR "Meningiomas, Malignant":ti,ab,kw OR "Meningiomas, Multiple":ti,ab,kw OR "Meningioma, Multiple":ti,ab,kw OR "Multiple Meningioma":ti,ab,kw OR "Multiple Meningiomas":ti,ab,kw OR

"Meningiomatosis":ti,ab,kw OR "Meningiomatoses":ti,ab,kw OR "Meningotheliomatous Meningioma":ti,ab,kw OR "Meningioma, Meningotheliomatous":ti,ab,kw OR "Meningiomas, Meningotheliomatous":ti,ab,kw OR "Meningotheliomatous Meningiomas":ti,ab,kw OR "Microcystic Meningioma":ti,ab,kw OR "Meningioma, Microcystic":ti,ab,kw OR "Meningiomas, Microcystic":ti,ab,kw OR "Microcystic Meningiomas":ti,ab,kw OR "Olfactory Groove Meningioma":ti,ab,kw OR "Groove Meningiomas, Olfactory":ti,ab,kw OR "Meningioma, Olfactory Groove":ti,ab,kw OR "Meningiomas, Olfactory Groove":ti,ab,kw OR "Olfactory Groove Meningiomas":ti,ab,kw OR "Papillary Meningioma":ti,ab,kw OR "Meningioma, Papillary":ti,ab,kw OR "Meningiomas, Papillary":ti,ab,kw OR "Papillary Meningiomas":ti,ab,kw OR "Parasagittal Meningioma":ti,ab,kw OR "Meningioma, Parasagittal":ti,ab,kw OR "Meningiomas, Parasagittal":ti,ab,kw OR "Parasagittal Meningiomas":ti,ab,kw OR "Posterior Fossa Meningioma":ti,ab,kw OR "Meningioma, Posterior Fossa":ti,ab,kw OR "Meningiomas, Posterior Fossa":ti,ab,kw OR "Posterior Fossa Meningiomas":ti,ab,kw OR "Psammomatous Meningioma":ti,ab,kw OR "Meningioma, Psammomatous":ti,ab,kw OR "Meningiomas, Psammomatous":ti,ab,kw OR "Psammomatous Meningiomas":ti,ab,kw OR "Secretory Meningioma":ti,ab,kw OR "Meningioma, Secretory":ti,ab,kw OR "Meningiomas, Secretory":ti,ab,kw OR "Secretory Meningiomas":ti,ab,kw OR "Sphenoid Wing Meningioma":ti,ab,kw OR "Meningioma, Sphenoid Wing":ti,ab,kw OR "Meningiomas, Sphenoid Wing":ti,ab,kw OR "Sphenoid Wing Meningiomas":ti,ab,kw OR "Wing Meningioma, Sphenoid":ti,ab,kw OR "Wing Meningiomas, Sphenoid":ti,ab,kw OR "Spinal Meningioma":ti,ab,kw OR "Meningioma, Spinal":ti,ab,kw OR "Meningiomas, Spinal":ti,ab,kw OR "Spinal Meningiomas":ti,ab,kw OR "Transitional Meningioma":ti,ab,kw OR "Meningioma, Transitional":ti,ab,kw OR "Meningiomas, Transitional":ti,ab,kw OR "Transitional Meningiomas":ti,ab,kw OR "Benign Meningioma":ti,ab,kw OR "Benign Meningiomas":ti,ab,kw OR "Meningioma, Benign":ti,ab,kw OR "Meningiomas, Benign":ti,ab,kw OR "Cerebral Convexity Meningioma":ti,ab,kw OR "Cerebral Convexity Meningiomas":ti,ab,kw OR "Convexity Meningioma, Cerebral":ti,ab,kw OR "Convexity Meningiomas, Cerebral":ti,ab,kw OR "Meningioma, Cerebral Convexity":ti,ab,kw OR "Meningiomas, Cerebral Convexity":ti,ab,kw OR [mh "Carcinoma"] OR "Carcinoma":ti,ab,kw OR "Carcinomas":ti,ab,kw OR "Epithelial Neoplasms, Malignant":ti,ab,kw OR "Malignant Epithelial Neoplasms":ti,ab,kw OR "Epithelial Neoplasm, Malignant":ti,ab,kw OR "Malignant Epithelial Neoplasm":ti,ab,kw OR "Neoplasm, Malignant Epithelial":ti,ab,kw OR "Neoplasms, Malignant Epithelial":ti,ab,kw OR "Epithelial Tumors, Malignant":ti,ab,kw OR "Epithelial Tumor, Malignant":ti,ab,kw OR "Malignant Epithelial Tumor":ti,ab,kw OR "Malignant Epithelial Tumors":ti,ab,kw OR "Tumor, Malignant Epithelial":ti,ab,kw OR "Epithelioma":ti,ab,kw OR "Epitheliomas":ti,ab,kw OR "Carcinoma, Undifferentiated":ti,ab,kw OR "Undifferentiated Carcinoma":ti,ab,kw OR "Undifferentiated Carcinomas":ti,ab,kw OR "Carcinoma, Anaplastic":ti,ab,kw OR "Anaplastic Carcinoma":ti,ab,kw OR "Anaplastic Carcinomas":ti,ab,kw OR "Carcinoma, Spindle-Cell":ti,ab,kw OR "Carcinoma, Spindle Cell":ti,ab,kw OR "Spindle-Cell Carcinoma":ti,ab,kw OR "Spindle-Cell Carcinomas":ti,ab,kw OR "Carcinomatosis":ti,ab,kw OR "Carcinomatoses":ti,ab,kw OR [mh "Neoplasms"] OR "Neoplasms":ti,ab,kw OR "Neoplasia":ti,ab,kw OR "Neoplasias":ti,ab,kw OR "Neoplasm":ti,ab,kw OR "Tumors":ti,ab,kw OR "Tumor":ti,ab,kw OR "Cancer":ti,ab,kw OR "Cancers":ti,ab,kw OR "Malignancy":ti,ab,kw OR "Malignancies":ti,ab,kw OR "Malignant Neoplasms":ti,ab,kw OR "Malignant Neoplasm":ti,ab,kw OR "Neoplasm, Malignant":ti,ab,kw OR "Neoplasms, Malignant":ti,ab,kw OR "Benign Neoplasms":ti,ab,kw OR "Neoplasms, Benign":ti,ab,kw OR "Benign Neoplasm":ti,ab,kw OR "Neoplasm, Benign":ti,ab,kw : 214,020 results

#4. #2 AND #3. 272 results

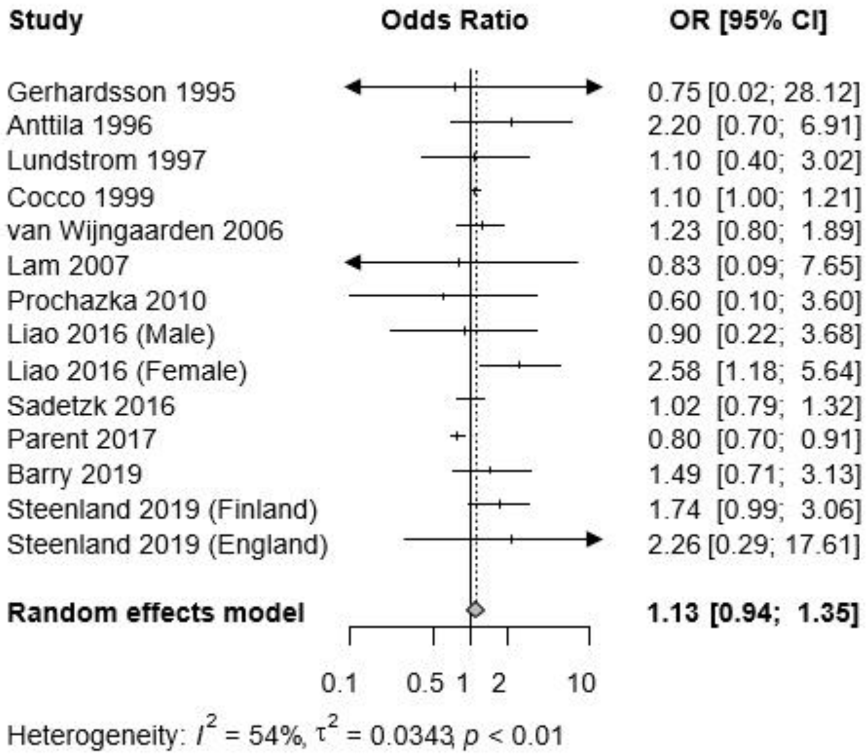

Figure S1. Forest plot of the studies about the association between occupational lead exposure and all brain tumor after excluding studies with high risk of bias

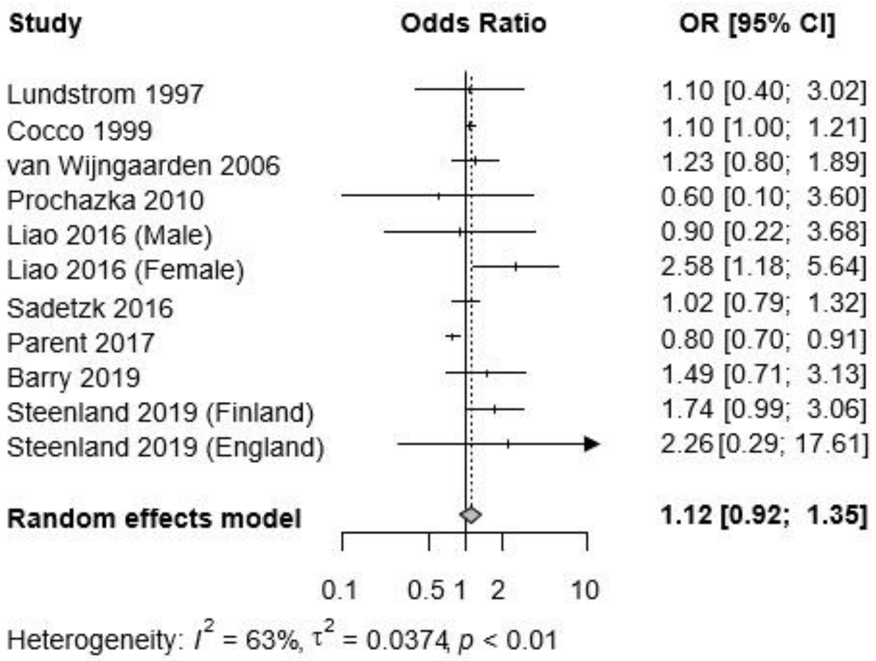

Figure S2. Forest plot of the studies about the association between occupational lead exposure and all brain tumor after excluding studies with medium and high risk of bias.

(a)

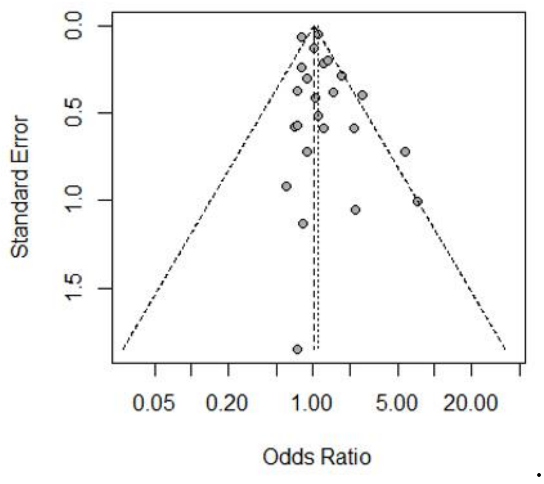

(b)

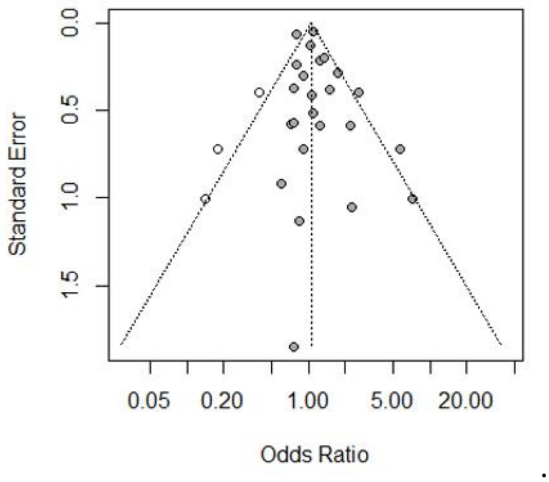

Figure S3. Funnel plot for the studies evaluating the association between occupational lead exposure and all brain tumors, before (a) and after imputing missing studies with trim and fill method (b).

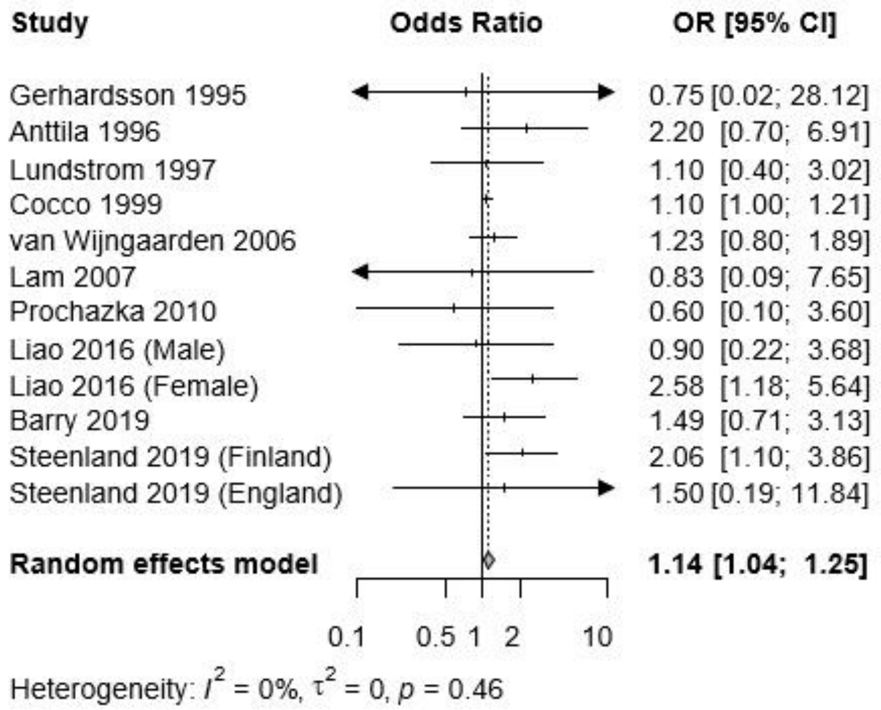

Figure S4. Forest plot of the studies about the association between occupational lead exposure and malignant brain tumor after excluding studies with high risk of bias

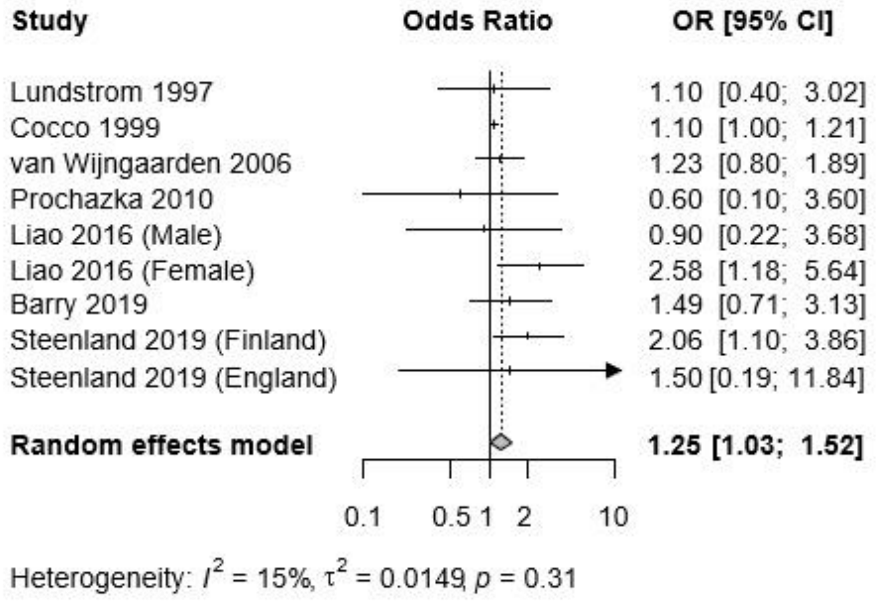

Figure S5. Forest plot of the studies about the association between occupational lead exposure and malignant brain tumor after excluding studies with medium and high risk of bias

.

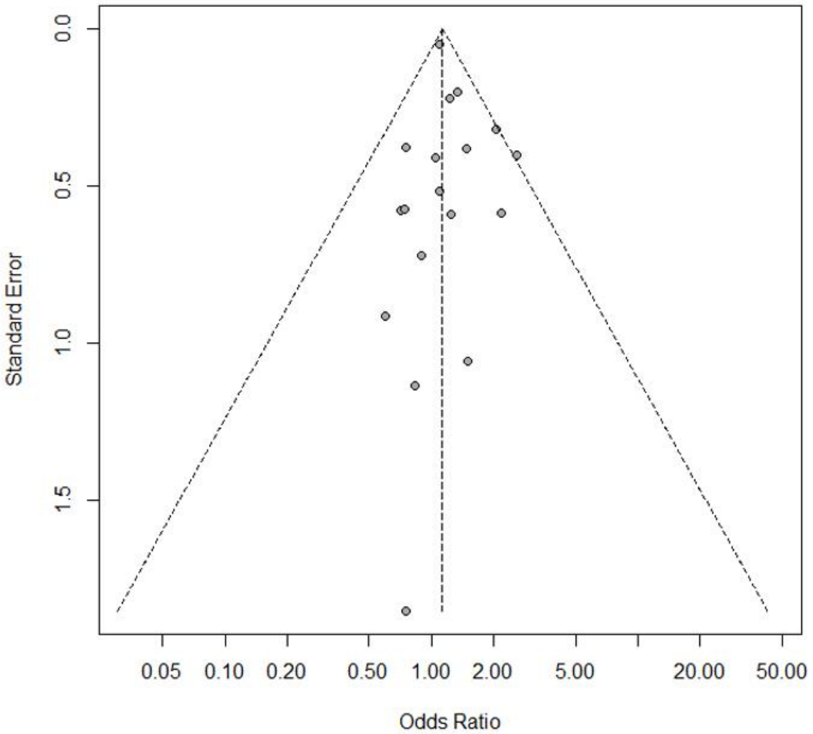

Figure S6. Funnel plot for the studies evaluating the association between occupational lead exposure and malignant brain tumors (the trim and fill method did not impute any missing studies).

Table S1. Subgroup analyses according to the tumor subtypes and sex.

Subgroup	No. of Studies	Pooled OR	Heterogeneity	
**Brain tumor subtype**			I^2, %	*p* value
Glioma	5	1.03 (0.67-1.57)	55	0.06
Meningioma	6	1.69 (1.02-2.79)	67	< 0.01
Subgroup difference: p = 0.1419				
**Sex**				
Female	7	1.23 (0.92-1.65)	52	0.05
Male	12	1.08 (0.86-1.35)	0	0.86
Subgroup difference: p = 0.4781				
